# Corneal Infections Associated with Sleeping in Contact Lenses — Six Cases, United States, 2016–2018

**DOI:** 10.15585/mmwr.mm6732a2

**Published:** 2018-08-17

**Authors:** Jennifer R. Cope, Nuadum Muriel Konne, Deborah S. Jacobs, Deepinder K. Dhaliwal, Michelle K. Rhee, Jia Yin, Thomas L. Steinemann

**Affiliations:** ^1^Division of Foodborne, Waterborne, and Environmental Diseases, National Center for Emerging and Zoonotic Infectious Diseases, CDC; ^2^Massachusetts Eye and Ear Infirmary, Boston, Massachusetts; ^3^University of Pittsburgh Medical Center Eye Center, Pittsburgh, Pennsylvania; ^4^New York Eye and Ear Infirmary of Mount Sinai, New York, New York; ^5^MetroHealth Medical Center, Cleveland, Ohio; ^6^Case Western Reserve University School of Medicine, Cleveland, Ohio.

## Abstract

Contact lenses, when worn and cared for properly, are a safe and effective form of vision correction used by an estimated 45 million Americans. However, contact lens wearers are at risk for contact lens–related eye infections, especially when wearers do not practice proper contact lens wear and care habits. These infections, affecting the cornea and known as microbial keratitis (Figure), can lead to serious adverse health outcomes. Because contact lenses are regulated by the Food and Drug Administration (FDA) as medical devices, contact lens–related corneal infections should be reported to FDA as an adverse event. To illustrate their serious health implications, six cases of contact lens–related corneal infection, in which sleeping in lenses was reported as the main risk factor, are presented. Consequences of infection reported among the identified cases included the need for frequent administration of antibiotic eye drops, multiple follow-up medical appointments, and permanent eye damage. Health education measures directed toward contact lens wearers should emphasize raising awareness of the risks of sleeping in contact lenses as well as adherence to all recommendations for the wear and care of contact lenses. Additional measures are needed to educate eye care professionals about the need to report contact lens–related corneal infections to MedWatch, the FDA Safety Information and Adverse Event Reporting program (https://www.fda.gov/MedWatch/).

**FIGURE:**
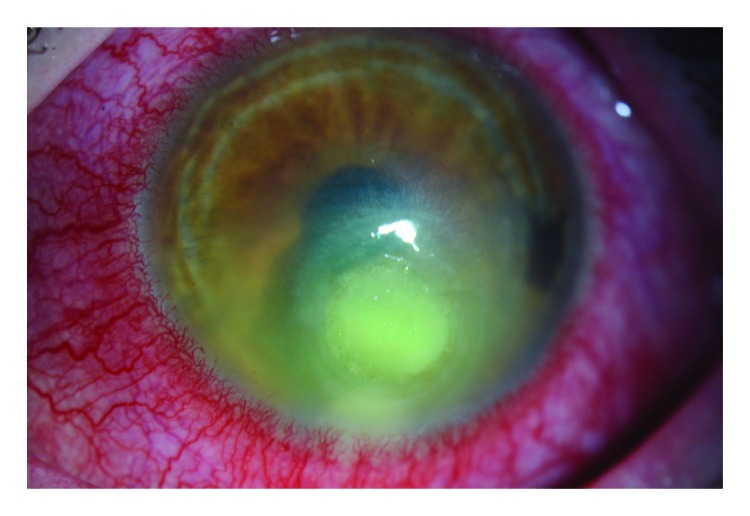
Findings characteristic of a contact lens–related corneal infection* Photo/Deborah S. Jacobs, Jia Yin * There is moderate injection, a notable paracentral white opacity with overlying ulceration, and surrounding haze.

Outside of MedWatch, no formal surveillance for contact lens–related corneal infections exists in the United States; in 2010, an estimated 1 million outpatient and emergency department visits were reported for keratitis of all types (*1*). Despite this high estimated annual prevalence, over an 11-year period, only 1,075 reports of contact lens–related corneal infections were reported to FDA’s MedWatch database (*2*). Among the many behaviors that increase the risk for a contact lens–related corneal infection, sleeping in lenses is one of the riskiest and one of the most commonly reported behaviors among adolescent and adult contact lens wearers (*3*). Approximately one third of contact lens wearers report sleeping or napping in their lenses. Sleeping in lenses, whether inadvertently, occasionally, or as part of a prescribed wearing schedule (i.e., extended wear lenses), increases the risk for contact lens–related eye infections six- to eightfold (*4*).

In collaboration with the Eye and Contact Lens Association (formerly known as the Contact Lens Association of Ophthalmologists), six cases of contact lens–related corneal infections were identified that were diagnosed in the last 2 years in which sleeping in lenses was reported as a risk factor. Patients were evaluated and treated by practicing ophthalmologists in four major academic medical centers. Clinical presentation, risk factors, treatment, and outcomes were reviewed.

## Case Reports

**Case 1.** A man aged 34 years with a 17-year history of soft contact lens use was evaluated for left eye redness and blurry vision. He reported sleeping in his contact lenses 3–4 nights per week and swimming with contact lenses. He was treated for bacterial and fungal microbial keratitis for 2 months without improvement. He was evaluated at an academic medical center, where confocal microscopy, a technique that provides serial images of sections through the cornea,* revealed findings suggestive of *Acanthamoeba* keratitis. He was treated with topical polyhexamethylene biguanide and chlorhexidine hourly that was tapered over 6 months. The infection resolved with final spectacle-corrected visual acuity of 20/40, requiring rigid contact lenses for correction to 20/20.

**Case 2.** A man aged 59 years wore his soft contact lenses overnight during a 2-day hunting trip and developed eye pain on the third day. He used over-the-counter eye drops with minimal response. On initial evaluation, he was diagnosed with a corneal abrasion and treated with a bandage contact lens to promote healing, along with tobramycin/dexamethasone drops prescribed four times daily. With worsening symptoms, his treatment was changed to ofloxacin drops every 2 hours. While in the shower, he wiped his eyes with a towel, then heard a popping sound and felt a painful sensation in his left eye. He was referred to ophthalmology where a large perforated corneal ulcer was diagnosed. An urgent corneal transplant was performed to reestablish the integrity of the eye, and he was treated with broad-spectrum topical antibiotics postoperatively. He recovered useful vision, which improved to 20/25 after cataract surgery 1 year later.

**Case 3.** A woman aged 34 years was evaluated for 3 days of sharp right eye pain. She routinely slept in her soft contact lenses and used lenses for longer than the recommended monthly replacement schedule. She reported not seeing an eye care professional in years and refilling her contact lens prescription through an online contact lens retailer for at least 5 years. Examination of the right eye revealed a paracentral 1.5 mm infiltrate with surrounding edema and trace anterior chamber cells. Symptoms and signs were improved the day after treatment with topical moxifloxacin. She was instructed to continue moxifloxacin but failed to return for a 1-week follow-up appointment as instructed.

**Case 4.** A man aged 57 years was evaluated in the emergency department with bilateral reduced vision and eye pain. He reported wearing the same soft contact lenses continuously for approximately 2 weeks. He did not disinfect his lenses daily, slept in them on a regular basis, and did not replace them regularly. On examination, uncorrected visual acuity was light perception in the right eye and hand motion in the left eye. The right eye revealed a central corneal infiltrate and perforation of the cornea. The left eye revealed a central infiltrate with two infiltrates paracentrally and a hypopyon (leukocytes in the anterior chamber of the eye). He received a diagnosis of bilateral bacterial keratitis. Hourly fortified tobramycin and vancomycin drops were required for treatment. A corneal transplant was required to save the right eye. The left eye responded to topical therapy with visual acuity of 20/40 and a central stromal scar.

**Case 5.** An adolescent female aged 17 years who slept in a soft contact lens purchased without a prescription at a chain store developed a right corneal ulcer; a culture grew *Pseudomonas aeruginosa*. She was started on fortified tobramycin and vancomycin eye drops. Her vision was light perception in the right eye, and the cornea showed a central white dense ulcer, stromal infiltrates, and 0.5 mm hypopyon. On follow-up, her vision had improved to 20/100, pinhole to 20/60. She had a stromal scar with thinning.

**Case 6.** A man aged 18 years went to the emergency department with a 3-day history of pain, redness, light sensitivity, and tearing in his left eye. He had a 1-year history of wearing decorative soft contact lenses^†^ obtained at a local store without a prescription. He also reported sleeping in his lenses. He was given fluoroquinolone eye drops in the emergency department and subsequently was seen at a local eye clinic, at which time bacterial keratitis was suspected. His vision was 20/25 in the right eye and 20/50 in the left. His left eye showed moderate injection with a central ulcer, edema, and moderate inflammatory reaction. Cultures were obtained, and hourly fortified cephalosporin and aminoglycoside drops were prescribed. Follow-up cultures of the patient’s eye, his lenses, and lens case each yielded heavy growth of *Klebsiella pneumoniae* and *Pseudomonas aeruginosa*. Ten days later his symptoms were better; vision in the left eye had improved to 20/25, but a stromal scar remained.

## Discussion

This case series of contact lens–related corneal infections highlights the burden these infections place on contact lens wearers and the serious outcomes associated with them. All of the patients required treatment with antibiotic eye drops, sometimes requiring administration hourly for weeks or months. This finding is consistent with a previous analysis of administrative health care data, which indicated that 76% of keratitis patient encounters were associated with an antimicrobial prescription (*1*). Some of the patients described in this series sought care in an emergency department, where it is more costly to receive care (*1*). One patient was lost to follow-up care, suggesting possible complete resolution of disease, but also highlighting the challenge of complying with medical care by patients who might be busy with work, school, or household obligations. Two patients required surgery, and most were left with permanent eye damage or vision loss. Contact lens wearers are younger on average than nonwearers and bear a burden of disease despite being viewed as healthy.

Exam findings in these patients were indicative of active infection including stromal opacification, anterior chamber reaction, and hypopyon. Cultures and diagnostic testing identified various organisms, including *Pseudomonas aeruginosa* and *Acanthamoeba* spp., which might suggest contamination of contact lenses and supplies with tap water.

In three of the six cases, contact lenses were purchased without a valid prescription. In one case, they were decorative lenses, which are lenses that alter the appearance of the eye (e.g., change the color) but might not improve vision. Decorative lenses, similar to lenses that are prescribed for vision correction, are classified as medical devices. The sale of all contact lenses is regulated and should require a valid prescription from an eye care professional. In the United States, contact lens prescriptions are valid for only 1–2 years, depending on the state. Visits with an eye care professional to renew a prescription serve as opportunities for reeducation about safe contact lens wear and care practices.

Sleeping in contact lenses is one of the most frequently reported contact lens risk behaviors and one with a high relative risk for corneal infection (*3*,*4*). Sleeping in lenses has been shown to be a risk factor regardless of lens material and frequency, with even occasional overnight use conferring risk (*5*,*6*). Although some contact lenses are approved by FDA for overnight wear, the increased risk for infection is acknowledged by their classification as a Class 3 medical device, which includes medical devices with the greatest risk for harm such as intraocular lenses and implantable pacemakers. Postmarketing surveillance of drugs and devices is important to the health and safety of the general public. Whereas medical device manufacturers are required to report adverse events, not all adverse events come to the attention of manufacturers (*7*). Patients and physicians can fill this gap.

The findings in this report are subject to at least three limitations. First, cases were chosen by practicing ophthalmologists (who can perform eye surgery), and are likely referred cases of contact lens–related eye infections that are more serious and might require surgical intervention. Therefore, the cases presented here are not necessarily representative of the typical contact lens–related eye infection. Second, as a case series, there are no definitive statements that can be made regarding the association of the reported risk factors and the contact lens–related eye infections. Finally, the patients in the cases reported here might have had an innate susceptibility to developing an eye infection; other contact lens wearers with the same habits might be able to sleep in lenses without adverse outcomes.

Cases of contact lens–related infections, such as those described here, should be reported as adverse events to the FDA Safety Information and Adverse Event Reporting Program at http://www.fda.gov/MedWatch. The Eye and Contact Lens Association is working to promote contact lens safety for patients by encouraging eye care professionals and patients to voluntarily report contact lens–related eye infections to FDA. Using the data accumulated in the adverse event reporting program, contact lens stakeholders (industry, regulatory authorities, eye care professionals, and public health) can work together to determine what improvements can be made to contact lenses, care products, manufacturer guidelines, and labeling. Health education measures directed toward contact lens wearers should emphasize raising awareness of the risks of sleeping in contact lenses as well as adherence to all recommendations for the wear and care of contact lenses.

**Summary**

**What is already known about this topic?**

Sleeping in contact lenses increases the risk for contact lens–related eye infections by six- to eightfold. Approximately one third of contact lens wearers report sleeping or napping in their lenses.

**What is added by this report?**

This report of six contact lens–related corneal infections associated with sleeping in lenses demonstrates that corneal infections might require surgical intervention and result in corneal damage and possible permanent vision loss.

**What are the implications for public health practice?**

It is important that contact lens wearers follow their eye care professional’s recommendations for contact lens use, including use during sleep. Cases of contact lens–related infections should be reported as adverse events to the Food and Drug Administration’s MedWatch (http://www.fda.gov/MedWatch).

## Footnotes

* https://www.sciencedirect.com/science/article/pii/S0002939409004577?via%3Dihub.

† Decorative or cosmetic lenses are contact lenses that change the look of the eye but might not correct vision. These lenses can be daily disposable, soft daily, soft extended wear, or rigid gas permeable lenses.

## References

[1] Collier SA, Gronostaj MP, MacGurn AK, . Estimated burden of keratitis—United States, 2010. MMWR Morb Mortal Wkly Rep 2014;63:1027–30. 25393221 PMC5779494

[2] Cope JR, Collier SA, Srinivasan K, Contact lens–related corneal infections—United States, 2005–2015. MMWR Morb Mortal Wkly Rep 2016;65:817–20. 10.15585/mmwr.mm6532a2 27538244

[3] Cope JR, Collier SA, Nethercut H, Jones JM, Yates K, Yoder JS. Risk behaviors for contact lens–related eye infections among adults and adolescents—United States, 2016. MMWR Morb Mortal Wkly Rep 2017;66:841–5. 10.15585/mmwr.mm6632a2 28817556 PMC5657667

[4] Dart JK, Radford CF, Minassian D, Verma S, Stapleton F. Risk factors for microbial keratitis with contemporary contact lenses: a case-control study. Ophthalmology 2008;115:1647–54.e3. 10.1016/j.ophtha.2008.05.003 18597850

[5] Stapleton F, Edwards K, Keay L, et al. Risk factors for moderate and severe microbial keratitis in daily wear contact lens users. Opthalmology 2012;119:1516–21. 10.1016/j.ophtha.2012.01.052 22521083

[6] Sauer A, Meyer N, Bourcier T; French Study Group for Contact Lens–Related Microbial Keratitis. Risk factors for contact lens-related microbial keratitis: a case-control multicenter study. Eye Contact Lens 2016;42:158–62. 10.1097/ICL.0000000000000180 26219076

[7] Food and Drug Administration. Mandatory reporting requirements: manufacturers, importers, and device user facilities. Silver Spring, MD: US Department of Health and Human Services, Food and Drug Administration; 2015. https://www.fda.gov/MedicalDevices/DeviceRegulationandGuidance/PostmarketRequirements/ReportingAdverseEvents/default.htm

